# Novel deep intronic mutation in PLA2G6 causing early-onset Parkinson’s disease with brain iron accumulation through pseudo-exon activation

**DOI:** 10.1007/s10048-021-00667-0

**Published:** 2021-08-13

**Authors:** Chiara Cavestro, Celeste Panteghini, Chiara Reale, Alessia Nasca, Silvia Fenu, Ettore Salsano, Luisa Chiapparini, Barbara Garavaglia, Davide Pareyson, Ivano Di Meo, Valeria Tiranti

**Affiliations:** 1 Unit of Medical Genetics and Neurogenetics, Fondazione IRCCS Istituto Neurologico Carlo Besta, Milan, Italy; 2 Unit of Rare Neurodegenerative and Neurometabolic Diseases, Fondazione IRCCS Istituto Neurologico Carlo Besta, Milan, Italy; 3 Unit of Neuroradiology, Fondazione IRCCS Istituto Neurologico Carlo Besta, Milan, Italy

**Keywords:** PLA2G6, PLAN, NBIA, Early-onset Parkinson’s disease, Pseudo-exon activation, Deep intronic variant

## Abstract

*PLA2G6* is the causative gene for a group of autosomal recessive neurodegenerative disorders known as *PLA2G6*-associated neurodegeneration (PLAN). We present a case with early-onset parkinsonism, ataxia, cognitive decline, cerebellar atrophy, and brain iron accumulation. Sequencing of *PLA2G6* coding regions identified only a heterozygous nonsense variant, but mRNA analysis revealed the presence of an aberrant transcript isoform due to a novel deep intronic variant (c.2035-274G > A) leading to activation of an intronic pseudo-exon. These results expand the genotypic spectrum of PLAN, showing the paramount importance of detecting possible pathogenic variants in deep intronic regions in undiagnosed patients.

## Introduction

*PLA2G6*-associated neurodegeneration (PLAN) is a heterogeneous group of rare autosomal recessive neurodegenerative disorders caused by mutations in the *PLA2G6* gene [1]. This gene encodes for iPLA2β, a group VIA calcium-independent A2 phospholipase, involved in phospholipids metabolism essential for maintaining cell membrane integrity. Depending on age of onset and clinical features, PLAN can be classified in three subtypes, including infantile neuroaxonal dystrophy (INAD), atypical neuroaxonal dystrophy (ANAD), and PARK14 autosomal recessive early-onset Parkinson’s disease (EOPD). INAD and ANAD typically occurred in childhood, often associated with cerebellar cortical atrophy and iron deposition in the brain, a condition known as neurodegeneration with brain accumulation type II (NBIA2) [2]. Contrariwise, EOPD onset is in early adulthood, typically associated with dystonia, rapid cognitive decline, psychosis, dysarthria, and pyramidal tract signs [3]. However, it has been increasingly reported that PLAN can manifest with intermediate phenotypes partially matching those classically associated with this disorder, thus preventing the identification of a precise genotype–phenotype correlation [4–6].

In general, genetic disorders causative mutations have been prevalently identified in exons and in RNA donor or acceptor splice sites. However, despite next generation sequencing (NGS) has revolutionized genetic testing, a considerable proportion of patients with a clinical diagnosis for a recessive condition have only one heterozygous mutation, suggesting the presence of not detected deep intronic variations. Intronic point mutations can activate pseudo-exons, such as intronic sequences flanked by apparently good-to-consensus acceptor and donor-site signals that are never recognized by the splicing machinery [7].

Here, we report the first case of PLAN caused by a combination of nonsense and deep intronic variants in *PLA2G6* gene.

## Materials and methods

This study was approved by the Ethics Committee of the Besta Institute with an informed, written consent. For sequencing of *PLA2G6* transcript, PCR products were processed with Nextera XT DNA sample kit (Illumina) [8]. Real-time quantitative PCR (qPCR) was performed in a CFX-96 system (Bio-Rad) using iTaq Universal SYBR Green Supermix (Bio-Rad), with a primer pair specific for PLA2G6; the ACTB gene was used as reference. In silico predictors were used to assess the effect of variants on splicing (SpliceAI, HSF, NetGene2, NNSplice, varSEAK, MaxEntScan) [9]. The c.2035-274G > A variant was submitted to the Leiden Open Variation Database (DB-ID: PLA2G6_000182).

## Results

### Clinical reports

The patient is a 43-year-old lady born to non-consanguineous parents and with no family history of neurological diseases. Her symptoms started at age 32 years with gait ataxia and scanned speech, and, since age 36, a progressive asymmetric parkinsonian syndrome, with rigidity, hypokinesia, resting and postural tremor predominantly on the right, and cognitive decline. Response to L-Dopa was poor. She later developed behavioral abnormalities—irritability and occasional aggressiveness—treated with quetiapine. She progressively lost walking ability and developed dysphagia and severe akinesia.

At 43 years, examination revealed severe cognitive decline in memory, linguistic, frontal-executive, and visual-spatial skills; pursuit saccadization, vertical upward gaze palsy, marked hypomimia, almost absence of spontaneous speech, which was scanned and monotonous, dysphagia, and drooling; severely hypokinetic gait, with double support; marked axial and limb rigidity, with trochlea sign, and generalized akinesia; oromandibular and bilateral upper limb resting tremor; increased deep tendon reflexes; and bilateral Babinski sign.

**Fig. 1:**
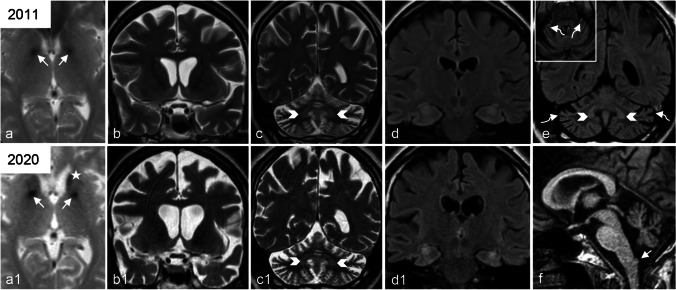
Neuroradiological findings**.** Brain MRI performed at age 34 (**a**–**d**) and age 43 (**a1**–**d1**, **e,** and **f**) showing typical PLAN neuroradiological findings, except for not atrophic optic chiasm. **a, a1** Axial T2 GRE images reveal, already in the first exam (**a**), globus pallidus hypointensity due to iron deposition (arrows); last examination (**a1**) demonstrates also iron in the head of the caudate nuclei (asterisk). **b, b1, c, c1** Coronal T2-wi demonstrate normal optic chiasm volume and progressive diffuse cerebral atrophy (**b** and **b1**), associated with mild dentate nucleus hyperintensity (arrowheads in **c**, **c1,** and **e**) and progressive cerebellar atrophy characterized by widening of cerebellar folia. **d, d1** Coronal FLAIR images show an unusual atrophy and hyperintensity of both hippocampi in the last exam compared to the first. **e** Coronal and axial FLAIR image demonstrate cerebellar cortical hyperintensity (curved arrows). **f** Midline sagittal T1-wi shows cerebellar vermian atrophy and clava hypertrophy (arrow)

Brain MRI showed T2 GRE hypointensity of the pallida, substantia nigra and head of the left caudate nucleus, cerebellar atrophy, T2 hyperintensity of cerebellar cortex and dentate nuclei, and clava hypertrophy, and diffuse cerebral atrophy with hyperintensity of the hippocampi (Fig. 1).

EEG showed some slow abnormalities in the temporal regions, more pronounced on the left side, photosensitivity, and photomyoclonic response, without clinical evidence of seizures. There was latency prolongation of the central components of somatosensory evoked potentials.

### Molecular investigations

**Fig. 2:**
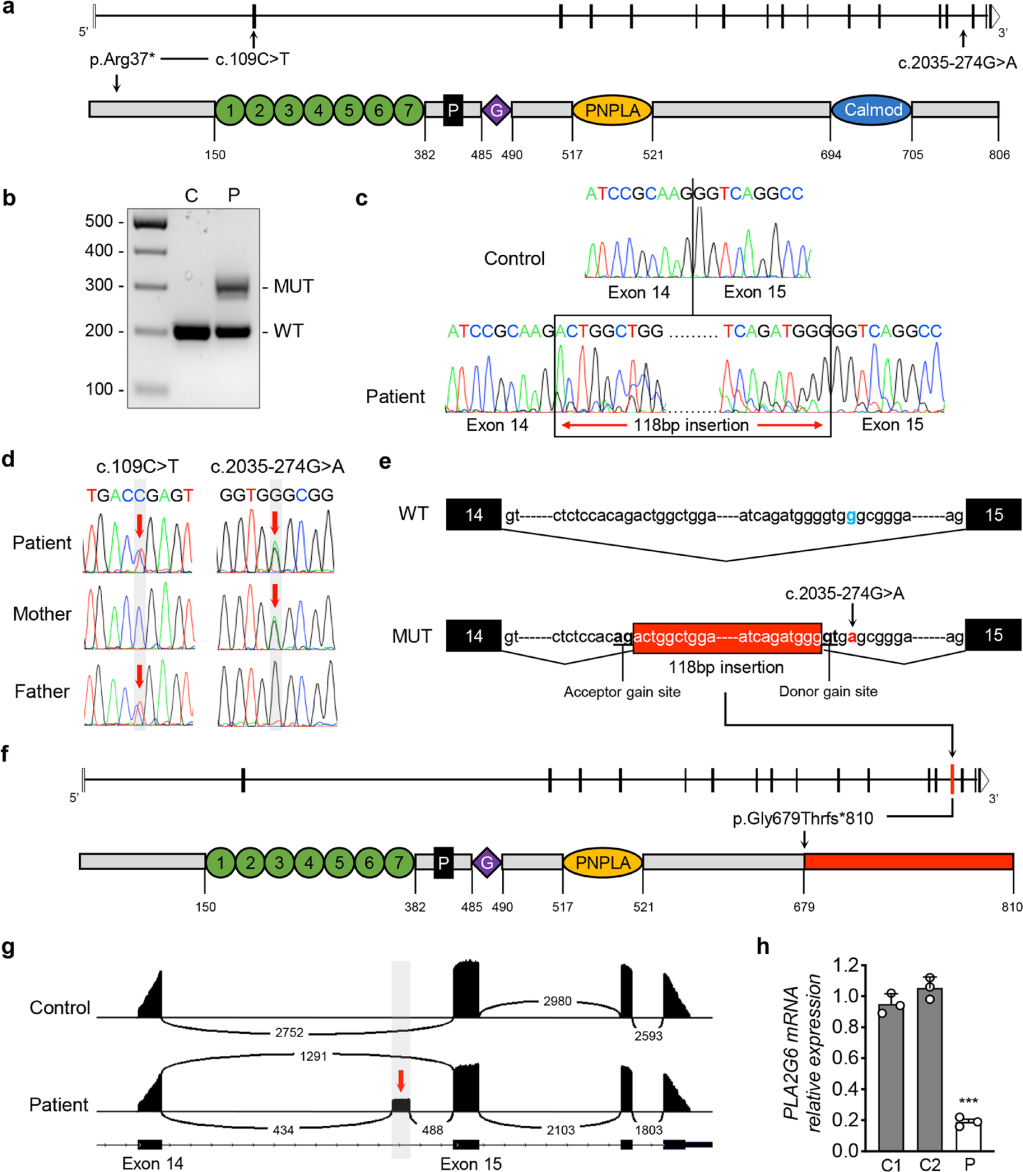
Molecular studies. **a** Schematic representation of the *PLA2G6* gene (upper) and protein (lower), and location of variants identified in this study. Protein consists of seven ankyrin repeats (numbered circles), a proline-rich motif (P), a glycine-rich nucleotide binding motif (G), a lipase motif (PNPLA), and a binding site for calmodulin (Calmod). Numbers shown below are the amino acid positions. **b** Amplicons spanning exons 14–15 of the patient (P) and a healthy control (C) show a different‐sized PCR product in the patient sample. **c** Sanger sequencing of *PLA2G6* cDNA shows inclusion of a 118-nucleotide intronic sequence between exons 14 and 15 (boxed) in patient. **d** Sanger sequencing of the *PLA2G6* gene show compound heterozygous variants. The c.109C > T variant is inherited from the father, the c.2035-274G > A variant is from the mother. **e** Schematic of *PLA2G6* exons 14–15 showing the c.2035-274G > A variant, which substitutes a less favored G (WT) at the + 4 position for a highly favored A (MUT), strengthens a naturally occurring cryptic donor splice site to activate spliceosomal inclusion of the intron 14 pseudo-exon (boxed region). **f** Schematic representation of PLA2G6 gene and protein resulting from pseudo-exon inclusion. **g** Sashimi plots of *PLA2G6* cDNA sequencing data show the presence of intron 14 pseudo-exon in patient, representing about 43% of total transcript. **h** Relative *PLA2G6* mRNA expression in control (C1, C2) and patient (P) fibroblasts. Mean of three independent experiments ± SD is shown. ***p < 0.001 (Student’s *t* test)

Sequencing of *PLA2G6* coding and flanking intron sequences revealed heterozygosity for the nonsense variant c.109C > T in exon 2 of the NM_003560.4 transcript (Fig. 2a), which introduces a premature stop codon at position 37 of the iPLA2β protein (p.Arg37*). This variant is reported as pathogenic in ClinVar database (accession: RCV000023318.6) and already associated with PLAN [10, 11]. However, we could find neither a second variant in the coding regions or adjacent introns nor the presence of exon deletion or duplication in *PLA2G6* by MLPA analysis. Therefore, we isolated RNA from patient’s fibroblasts and retrotranscribed it into cDNA to search for a second variant. Using intron-spanning primers pairs, we amplified an abnormal band of higher molecular weight in the patient that was not detected in controls (Fig. 2b). Sequencing of patient’s *PLA2G6* transcript revealed the inclusion of a 118 nt region belonging to intron 14 (Fig. 2c). Then, we performed Sanger sequencing to explore the noncoding regions flanking the included sequence and identified a heterozygous deep intronic variant at position chr22.hg19:38,509,935 (c.2035-274G > A) (Fig. 2d, e), which was absent in the Genome Aggregation Database (gnomAD). In silico algorithms predicted the c.2035-274G > A variant generates a pseudo-exon activation, gaining a novel splicing donor site 118 nt downstream of a preexisting cryptic acceptor site (Fig. 2e, Table 1). The pseudo-exon inclusion causes a frameshift mutation (p.Gly679Thrfs*810), leading the complete loss of calmodulin domain (Fig. 2f). Sequencing of parents’ genomic DNA confirmed that c.109C > T and c.2035-274G > A variants were transmitted from father and mother, respectively (Fig. 2d). By NGS, we found that the isoform with the pseudo-exon inclusion represents about 43% of patient’s total *PLA2G6* transcript (Fig. 2g). Likewise, the nonsense c.109C > T variant is present in about 55% of mRNA (not shown). Moreover, qPCR performed on cDNA from patient and age-matched control fibroblasts showed a reduction of about 80% in the expression level of *PLA2G6* transcript (Fig. 2h), suggesting that both variants lead to nonsense-mediated mRNA decay.

**Table 1:** Splicing predictions of c.2035-274G > A variant by multiple bioinformatic prediction tools

Prediction tool	Acceptor gain site		Donor gain site	
	Wild-type score	Mutant score	Wild-type score	Mutant score
SpliceAI	n.a	0.67	n.a	0.64
HSF	85.21	85.21	79.82	90.1
NetGene2	0.22	0.22	-	0.64
NNSplice	0.66	0.66	-	0.61
varSEAK	n.a	n.a	-48.06	+ 29.04
MaxEntScan	7.49	7.49	1.95	5.97

*n.a.* not available

## Discussion

It has been estimated that approximately half of the patients affected by rare genetic diseases remains without a definite molecular diagnosis, about 10% of which is due to pathogenic variants located deep within introns [7]. Here, we reported for the first time a deep intronic mutation in the *PLA2G6* gene, causing the creation of a new donor splice site leading to a pseudo-exon inclusion by activating a preexisting cryptic acceptor splice site. This case, in which only one coding variant was detected at first, highlights that the existence of putative dominant variants in *PLA2G6* should be reexamined [12]. Moreover, different studies reported that a consistent fraction of cases, ranging from 8 to 45%, were heterozygous for a single *PLA2G6* mutant allele, missing the second mutation [10, 13–15]. Our patient’s presentation is consistent with the rare phenotype observed in young adults, with atypical findings such as the T2 GRE hypointensity in the left caudate nucleus head and T2 hyperintensity in both hippocampi. Residual PLA2G6 activity may explain later symptom onset. To the best of our knowledge, the molecular mechanism here identified has never been described in genetic forms of Parkinson’s disease. Furthermore, this case underlines the importance of cDNA analysis for detection of mutations in the intronic sequence of candidate genes in instances where exon sequencing and MLPA fail to provide a conclusive diagnosis. Although whole genome sequencing is entering faster and faster in the clinical diagnostic, RNA analysis offers a valid alternative for selected cases, although it may present limitations linked to sample availability, tissue-specific gene expression, or mutations inducing complete nonsense-mediated mRNA decay.
